# Serum matters: human platelet lysate enables physiological modeling of HIV-1 infection in dendritic cells

**DOI:** 10.3389/fimmu.2025.1661425

**Published:** 2025-09-17

**Authors:** Paul Schweighofer, Magdalena Erlacher, Wilfried Posch, Doris Wilflingseder

**Affiliations:** ^1^ Institute of Hygiene and Medical Microbiology, Medical University of Innsbruck, Innsbruck, Austria; ^2^ Ignaz Semmelweis Institute, Interuniversity Institute for Infection Research, Vetmeduni, Vienna, Austria

**Keywords:** HIV-1, complement, xeno-free, human platelet lysate (HPL), dendritic cell

## Abstract

Monocyte-derived Dendritic Cells (moDC) are fundamentally involved in infectious processes and serve as model cell type for studying immune regulatory functions *in vitro*. How DCs behave in viral infections is influenced by various intra- and extracellular factors. Thus, working as physiologically as possible is crucially important for gaining mechanistic insights. We and others previously stressed a critical role of DCs in the course and transmission of human immunodeficiency virus (HIV) infection. Up to date, moDCs are differentiated in presence of FCS-based media, comprising undefined factors that might impact cell differentiation behavior and function. While a few studies addressed working under xeno-free conditions enhancing reproducibility and clinical applicability, differences between FCS and hPL-generated DCs with respect to infection disease processes remain poorly defined. In this study we established an animal component-free protocol to induce primary moDCs, and systematically compared FCS stimulated DCs versus hPL-induced DCs phenotypically and functionally. Our data revealed, that hPL shows high differentiation potential and infection potency of DCs by complement-opsonized HIV-1 (HIV-C). All in all, we established an animal component-free *in vitro* model to generate primary CD11c^+^CD209^+^-moDCs by use of hPL, which is a valuable tool to study viral infections and interactions *in vitro* and under more defined conditions compared to FCS-generated DCs.

## Introduction

Dendritic cells (DCs) are a heterogenous population of antigen presenting cells (APCs) (1) and major players in directing either immunity or tolerance (2, 3). Human DCs derived from classical CD14^+^ peripheral blood monocytes (moDCs) are one of the most common used approaches to generate large numbers of DCs for the investigation of inflammatory and infectious processes but also for therapeutic purposes, i.e. vaccine testing (4) or immunoregulatory cell therapies (5).

The role for DCs in controlling viral infections has been demonstrated by numerous studies, owing to their great relevance of immunomodulatory function and mechanism of action against pathogens (6). Research on the interaction between human immunodeficiency virus (HIV) infection and DC evidenced their susceptibility *in vitro* (7), in line with the substantial decline of myeloid and plasmacytoid dendritic cell (DC) subsets in the peripheral blood with increase in plasma viral load of chronic HIV patients (8). Thus, understanding the pathogenic and protective function of DCs during infection is necessary for ameliorating antiviral approaches.

MoDCs represent a well-defined DC subset mainly marked by the expression of key characteristic marker CD209 (DC-SIGN), which has been shown to recognize and bind many pathogens, including HIV-1 (9) and is specifically regulated in moDCs by activation signals (10). Additionally, moDCs are characterized by typical DC markers (i.e. expressing CD11c, CD1a, CD11b) and downregulate CD14 (11). These cells possess high potency to synthesize pro-inflammatory cytokines (3), and strongly resemble DCs found *in vivo* under inflammation.

Typically, DCs are generated in the presence of fetal calf/bovine serum (FCS/FBS), granulocyte/macrophage-colony stimulating factor (GM-CSF) and interleukin-4 (IL-4) (12–14). FCS is comprised of various components to supply cells with factors promoting proliferation and differentiation, i.e. growth factors, hormones, proteins, trace elements and vitamins (15). However, using bovine sera as cell culture supplement leads to several challenges in terms of safety (i.e. xenogeneic, risk for viral or prion transmission) and reproducibility due to inconsistency of serum batches, especially in clinical oriented studies, or specific cell-culture based applications. Moreover, a detrimental role for FCS has previously been shown in influenza virus growth (16, 17) and in hepatitis C virus (HCV) infection (18). However, the role of FCS in the regulation of HIV remains largely unknown.

With the aim to minimize animal-based research (3R principle) and to reduce variations impacting cell growth, morphology and functionality, we investigated whether a xenogeneic-free media supplement, human platelet lysate (hPL), can retain full DC differentiation potential and immunomodulatory properties compared to FCS-derived DCs. Moreover, we investigated the infection potential of DCs by use of complement-opsonized HIV-1 (HIV-C) and non-opsonized HIV-1 (HIV-1) in the various culture conditions. We demonstrated that animal component-free culture derived DCs are phenotypically and functionally similar to FCS-DCs and describe a novel *in vitro* model allowing further study of physiological responses in infectious diseases.

## Results

### Immunophenotypic comparison of FBS and hPL generated primary moDCs

**Figure 1:**
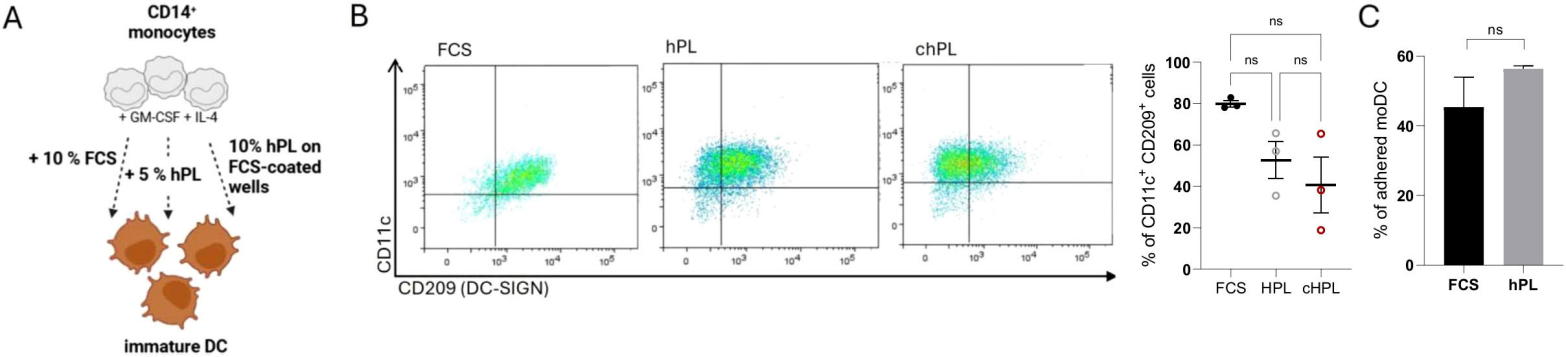
**(A)** Schematic overview of *in vitro* moDC generation from primary CD14^+^ peripheral blood monocytes. The figure was generated using BiorenderTM (license owned by Medical University of Innsbruck). **(B)** CD14^+^ monocytes were stimulated with 200 U/ml IL-4 and 250 U/ml GM-CSF grown in either FCS-based medium or hPL-based medium with or without FCS-pre-coating. On day 6, cells were harvested and analyzed for expression of CD11c and CD209 (DC-SIGN). (n= 3± SEM, 1-way ANOVA, corrected with Tukey multiple comparison test, *p<0.05). **(C)** Graph represents the percentage of adherent cells cultivated presence of hPL or FCS. (n = 3± SEM, 2-tailed Student *t* test, *p<0.05).

Several publications previously evidenced the efficient generation of monocyte-derived DCs (moDCs) upon stimulation with GM-CSF and IL-4 in FCS-supplemented media (19). However, recent reports demonstrated that different DC subsets can be generated without the addition of FCS, ensuring xeno-free cultivation conditions and facilitating clinically oriented studies (20, 21). Therefore, we first aimed to phenotypically compare primary generated DCs cultured under different serum-supplemented conditions, specifically in presence of 10% FCS (FCS), 5% hPL (hPL) or 1% FCS-pre-coated wells with 5% human platelet lysate (chPL) supplemented RPMI media (as previously published by Rauch et al. (22) (**Figure 1A**). Human peripheral blood CD14^+^ monocytes were purified by magnetic bead separation as previously described (19) and stimulated with GM-CSF and IL-4 to induce DC differentiation. On day 6, DCs were analyzed for their expression of moDC-affiliated markers, i.e. CD11c and CD209 (**Figure 1B**). Flow cytometric analysis across all tested donors confirmed that monocytes differentiated in hPL-supplemented media display similar FSC versus SSC identification and gain DC-characteristic CD11c and CD209 positivity as cells cultivated in FCS-containing or FCS-coated media (**Figure 1B**). In parallel, no cell morphologic differences could be observed, albeit moDCs in the presence of hPL showed an increased number of adherent cells maintaining DC-like morphological characteristics (**Figure 1C**).

### Upregulation of maturation markers on HIV-infected moDCs

**Figure 2:**
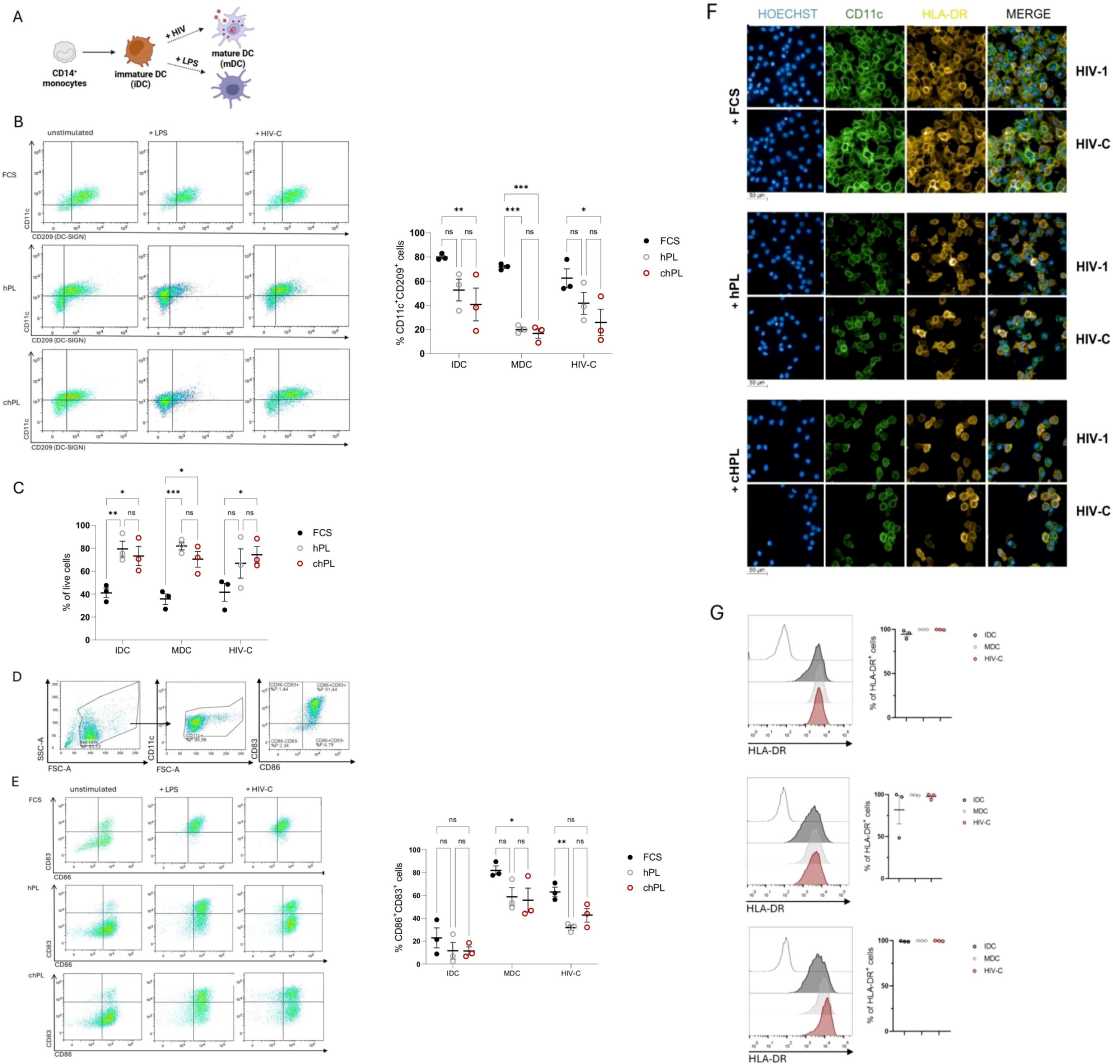
**(A)** Schematic overview of human primary monocyte derived DCs (iDCs) stimulated with bacterial or viral stimuli to induce maturation (mDCs). The figure was generated using BiorenderTM (license owned by Medical University of Innsbruck). **(B)** CD14^+^ monocytes were stimulated with 200 U/ml IL-4 and 250 U/ml GM-CSF grown in either FCS-based medium or hPL-based medium with or without FCS-pre-coating. Immature cells were stimulated for 24h with either 100 ng/ml LPS or 50 ng/ml HIV-C. On the left a representative dot plot of CD11c and CD209 (DC-SIGN) expression is depicted of unstimulated iDCs, LPS-stimulated mDCs (+LPS) or HIV-C-stimulated DCs (+HIV-C), on the right the summary of three independent donors of % CD11c/CD209 double-positive DCs is shown (n= 3± SEM). A 1-way ANOVA with Tukey´s multiple comparison test to evaluate effects of FCS, hPL or chPL on DC phenotype was performed, with *p<0.05, **p<0.01, ***p<0.001, ****p<0.0001. **(C)** Graph shows the survival of cells (% of viable cells) post infection depending on the growth medium and the treatment. 3 biological replicates were performed and % of live cells was statistically evaluated using GraphPad prism. A 1-way ANOVA with Tukey´s multiple comparison test to evaluate effects of FCS, hPL or chPL on DC phenotype was performed, with *p<0.05, **p<0.01, ***p<0.001, ****p<0.0001. **(D)** Representative dot plots of the gating strategy used to classify activated CD83^+^CD86^+^moDCs. Firstly, FSC-A vs. SSC-A dot plot and doublets were excluded. Immune cells were analyzed for DC marker CD11c. CD11c^+^ cells were finally gated for double expression of CD83 and CD86. **(E)** Immature moDCs were stimulated for 24h with either 100 ng/ml LPS or 50 ng/ml HIV-C and analyzed for the expression of CD83 and CD86. (n= 3± SEM, 1-way ANOVA, corrected with Tukey multiple comparison test, *p<0.05). On the left a representative dot plot of CD83 and CD86 expression is depicted of unstimulated iDCs, LPS-stimulated mDCs (+LPS) or HIV-C-stimulated DCs (+HIV-C), on the right the summary of three independent donors of % CD86/CD83 double-positive DCs is shown (n= 3± SEM). A 1-way ANOVA with Tukey´s multiple comparison test to evaluate effects of FCS, hPL or chPL on DC phenotype was performed, with *p<0.05, **p<0.01, ***p<0.001, ****p<0.0001. **(F)** Immature moDCs were stimulated for 24h with either 100 ng/ml LPS (not depicted), 50 p24 ng/ml of non-opsonized (HIV-1) or opsonized or(HIV-C) HIV-1. Representative staining of the DC phenotype (CD11c in green, HLA-DR in yellow) and infection by confocal microscopy are illustrated. Nuclei were visualized with Hoechst. Scale bars 50µm (underneath the pictures). Experiments were performed three times in independent biological replicates and counting at least 200 cells per condition. **(G)** Representative FACS plot and analysis of immature moDCs stimulated for 24h with either 100 ng/ml LPS, 50 ng p24/ml HIV-C to monitor the percentage of HLA-DR^+^ cells. (n= 3± SEM (biological replicates)).Data were analyzed using 1-way ANOVA, corrected with Tukey multiple comparison test. No significant differences between conditions (iDC, mDC, HIV-C-DC) were detected.

Depending on microenvironmental factors and functional necessity, monocytes and DCs possess high plasticity and can rapidly adopt phenotypically and functionally. Hence, moDCs are crucially implicated in inflammation and play a pivotal role during infection, i.e. HIV infection (23–26). Thus, we next sought to evaluate whether hPL-generated DCs provide a suitable model to study infectious diseases. The expression pattern of characteristic DC surface markers (CD83, CD86, CD11c and DC-SIGN) on complement-opsonized HIV-1-exposed (HIV-C) DCs was studied by FACS analyses and compared with that of LPS-treated DCs (mature DCs, mDCs) or untreated DCs (immature DCs, iDCs) (**Figure 2A**). Interestingly, loss of CD11c expression in DCs upon HIV-C exposure was consistently observed in all culture conditions, resembling an abundantly described key characteristic in HIV-infected patients (23) (**Figure 2B**). Moreover, as observed by Date et al. (21), hPL-grown DCs showed consistent elevated survival post infection, when compared to FCS-stimulated DCs (**Figure 2C**). Following, activation markers were monitored on FCS-, hPL- and chPL DCs in unstimulated control DCs (iDCs). The gating strategy of DC activation for flow cytometry assay is shown in **Figure 2D**. As expected, stimulation with LPS led to a consistent transition into mature DCs evidenced by increased levels in co-stimulatory molecules CD83 and CD86 levels. Similarly, exposure to HIV-C or LPS induced upregulation of CD83 or CD86 in all cultivation conditions (**Figure 2E**), although a much stronger maturation effect could be observed in FCS-supplemented condition. Moreover, in line with Švajger et al. (20), CD83 expression of iDCs remained low in all tested conditions, while CD86 levels were consistently higher in conditions using hPL. Finally, DC maturation was analyzed by confocal microscopy. As shown in in **Figure 2F**, DCs differentiated from monocytes using FCS revealed higher cell density compared to hPL and chPL-DCs. However, immunofluorescence staining, and flow cytometric analysis revealed positivity of HLA-DR under all tested conditions (**Figures 2F, G**). Consistent with Švajger et al. (20), the expression of activation markers was slightly lower in hPL conditions (**Figure 2F**). Thus, all additives possess capacity to induce a full mature DC phenotype by activating iDCs (LPS, HIV-C). The loss of CD11c expression in infected hPL-DCs might reflect the DC functionality *in vivo* (**Figure 2F**).

### hPL generated moDCs represent a potent animal-component free infection model to study HIV infection

DCs are crucially implicated in virus capture and transfer process, specifically representing a main potential target for HIV-1 transmission (23). Therefore, full functionality of the variously differentiated DC cultures is fundamental for studying infectious diseases *in vitro*.

In a next step, we further elucidated the HIV infection ability of DCs by use of confocal microcopy. As in the initial infection phase, transferred HIV virions are opsonized by complement proteins (i.e.C1q or C3), enhancing viral uptake (27, 28), we compared the infection potency and distribution of complement-opsonized HIV (HIV-C) and non-opsonized HIV-1 (HIV-1) in FCS- and hPL DCs.

**Figure 3:**
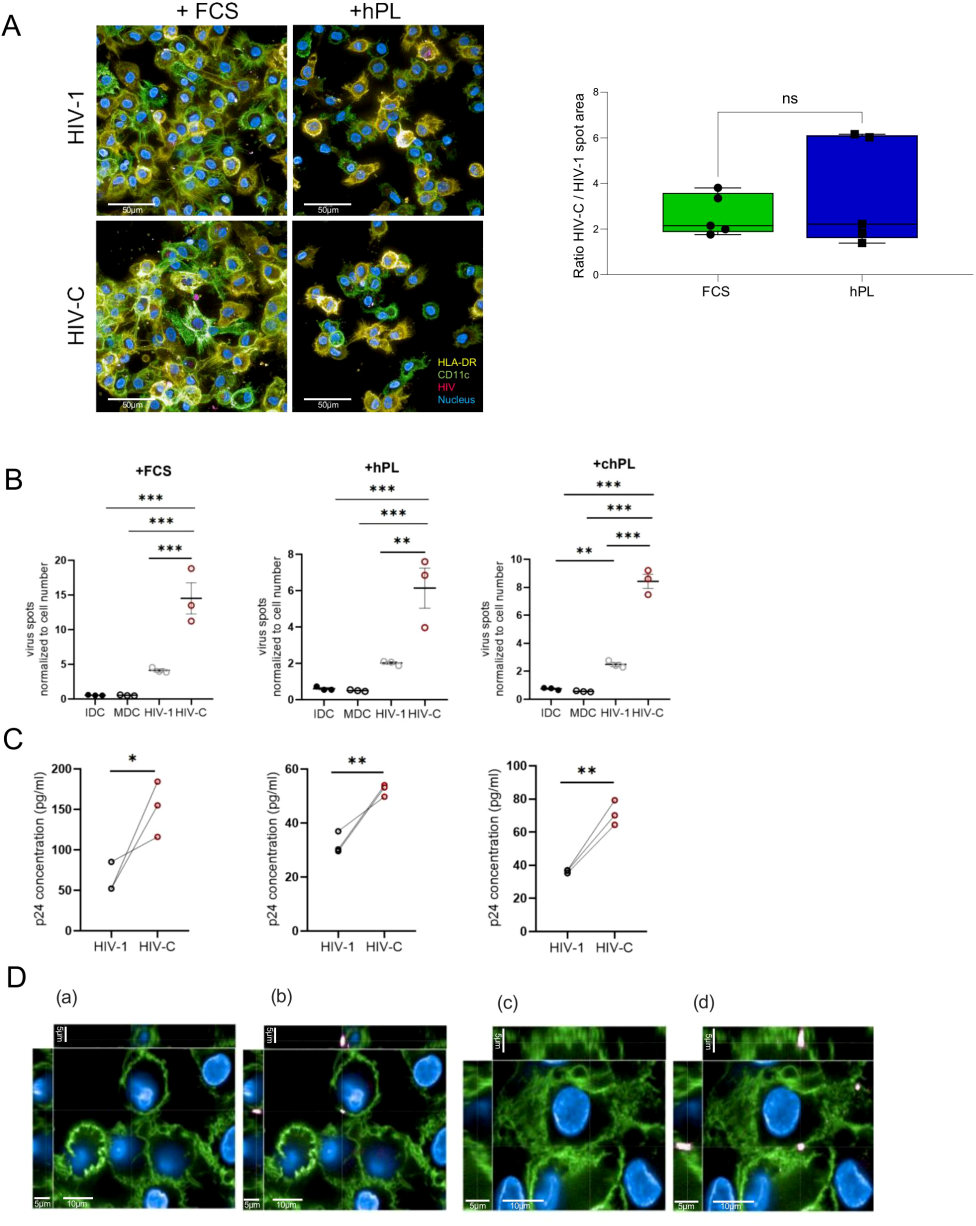
**(A)** (Left) Representative image of infection analysis of moDCs via confocal microscopy investigation of mCherry-HIV levels. MoDCs grown under FCS or hPL conditions were treated for 24h with either 50 ng/ml HIV-1 or HIV-C. Cells were stained for HLA-DR (yellow), CD11c (green), nuclei (blue) and virus (pink). Overlays are depicted. (Right) Virus areas were quantified using the Harmony 4.8 software (Perkin Elmer) in five independent spots for HIV-1 or HIV-C-exposed DCs and ratios were calculated using GraphPad prism. No significant differences were observed. At least 200 cells per condition were evaluated. Enhancement of infection in HIV-C- compared to HIV-DCs was observed in FCS- and hPL conditions. **(B)** Quantification of virus particles normalized to the cell number in moDCs grown under FCS, hPL or chPL condition. (n= 3± SEM, 1-way ANOVA, corrected with Tukey multiple comparison test, *p<0.05, **p<0.01, ***p<0.001). **(C)** Detection of HIV-1 p24 antigen via quantitative p24 ELISA in moDCs grown under FCS, hPL or chPL condition stimulated with either 100 ng/ml LPS, 50 p24 ng/ml HIV-C or HIV-1. Measurements have been performed 210 min after substrate addition. The signal measurement is performed at λ = 550 nm. The signal for the background is measured at λ = 650 nm. For normalization, the blank value is deducted from the signals. **(D)** Cellular localization of virus particles between HIV-1 and HIV-C. DCs were stained for the expression of CD11c and the mCherry tagged virus particles. (a) illustrates pocket formation without m-cherry-channel in HIV-1 treated moDCs. (b) shows pocket formation with the mCherry-channel switched on in HIV-1 treated moDCs. (c) depicts co-localization of CD11c and HIV-C without m-cherry-channel turned on in HIV-C treated moDCs. (d) shows co-localization of CD11c and HIV-C with mCherry-channel depicted in HIV-C treated moDCs. These were common staining patterns across all conditions (FCS, hPL, chPL) and multiple cells.

Interestingly, a higher cell number was displayed in the FCS supplemented media (**Figure 3A**). Immunohistology data of mCherry-tagged complement-opsonized HIV or HIV-1 (pink), HLA-DR (yellow) and CD11c (green) revealed successful viral entry in FCS and hPL culture conditions (**Figure 3A**, left). Expectantly, increased virus particle areas were consistently observed in HIV-C infections compared to HIV-1 infections as depicted by ratios of HIV-C versus HIV-1 spot areas (**Figure 3A**, right). When quantifying the virus particles normalized to the cell number, significantly higher virus spots upon HIV-C stimulation could be observed in all tested conditions (**Figure 3B**, FCS–left, hPL–middle, chPL-right). To further confirm that these cells were productively infected and secrete infectious virus particles, supernatants were collected, and infectivity was measured. The HIV p24 antigen, as representing the most abundant HIV protein is clinically used to detect early HIV infection (29). Differentially cultivated DCs were infected and an ELISA setup targeting the HIV-1 capsid protein p24 was performed. Despite the generally higher p24 concentration levels in the FCS moDC culture, stimulation with HIV-C significantly upregulated p24 levels superseding HIV-1 in all DC conditions (**Figure 3C**). Finally, we assessed the difference in plasma membrane localization of virus particles between HIV-1 and HIV-C. **Figure 3D** represents a XYZ-analysis of HIV-1 and HIV-C upon uptake in DCs. Co-localization of HIV with CD11c was only observed when DCs were exposed to HIV-C independent on the culturing method (FCS, hPL) (**Figure 3D**), whereas non-opsonized HIV-1 did not accumulate in CD11c-rich fractions.

## Discussion

In this study, different media additives were evaluated as animal component-free alternative approach for the *in vitro* generation of moDCs, to reduce animal-based research and to enable investigating infections under reliable physiological conditions. We showed that moDCs can be efficiently generated under hPL-supplemented conditions, expressing similar immunophenotypic characteristics when compared to FCS-derived moDCs. Moreover, we demonstrated that hPL differentiated moDCs are fully functionable in terms of DC maturation upon antigenic stimulation and provide a suitable model for studying infectious diseases, i.e. HIV. MoDCs serve as model cell type to study various inflammatory associated diseases or infections. Protocols for the efficient generation of moDCs mostly rely on the addition of FCS. However, FCS-conditioned medium contains numerous undefined factors that might affect differentiation behavior and function, thus leading to potentially distorted results. Moreover, FCS is potentially immunologically dominant, which can cause anaphylactic reactions. Thirdly, in consideration of the 3Rs principle (Refinement, Reduction, Replacement) and the animal use in research, further progress in valuable alternatives will be clinically, ethically and economically useful (30).

For the initial validation, we differentiated moDCs in various media supplements (hPL; FCS, hPL on FCS-coated wells to significantly reduce the FCS amounts needed in cell culture). Firstly, cell morphological changes were assessed (data not shown). As displayed herein, characteristics of immature moDCs are evident among the three conditions. All supplemented cultures led to efficient generation of CD11c^+^CD209^+^ moDCs. Although the total expression of CD11c was lower on moDCs in hPL-supplemented medium, their fluorescence intensity of CD209 was comparable with moDCs differentiated using FCS. In line with Švajger (20) we observed an increase in adherent cells in the hPL conditioned cells. Matured adherent vs. non-adherent DCs have been analyzed previously (31) showing increased expression levels of maturation markers (MHCII, CD83, CD86) and significantly higher DC purity on adherent cells compared to the non-adherent cell fraction. Thus, it would be interesting to investigate, whether increased proportion of adherent cells correlates with the expression of CD209 in hPL conditioned medium. Moreover, in this study 5% of hPL was used for the differentiation. Whether different hPL and FCS concentrations lead to differences in the marker expression profile and adherence of cells needs to be further investigated.

In the context of DC maturation, DC maturation markers (HLA-DR, CD83, CD86) were induced in all cell culture condition upon bacterial (LPS) and viral (HIV-1) stimulation. However, we found that moDCs generated in the presence of FCS gain higher levels of CD83 and CD86. This observation was previously reported by Date et al. (21), who showed IFN treatment of hPL IFN-DCs results in less pronounced up-regulation of particularly CD83 compared to FCS. Our notion that immature FCS-moDCs display higher levels of HLA-DR is reminiscent of a previous report, which described a concentration dependency of FCS and HLA-DR expression in the pro-monocytic cell line U937 (J. 32). Thus, it remains possible that this effect might be attributed to FCS. However, for further conclusions a broader DC marker profile and various DC activation stimuli need to be evaluated. Moreover, to exclude that above-described effects are not serum concentration dependent, testing of different FCS and hPL concentration is necessary. In future studies it would be interesting to address whether hPL allows similar cell plasticity for the generation of tolerogenic DCs. To fully functionally characterize hPL-moDCs a comprehensive T-cell polarization profile is needed.

We here showed that hPL represents a suitable differentiation model to study infectious diseases, in particular HIV. The observed rapid decline of CD11c expression upon stimulation with HIV or LPS has been observed in all moDCs. *In vivo* data are consistent with this observation, where CD11c expression is drastically decreased upon cell activation with poly I:C and LPS in mouse BMDC (33), or where loss of blood CD11c^+^ DCs in chronic HIV patients has been reported (8). Progressive infection examines the potential of cells to infect neighboring cells with self-generated virus particles. With non-complement opsonized HIV-1 as a factor for comparison of the potential of complement-opsonized HIV-C in the different cultivation conditions, a significant rise in virus production of HIV-C infected cells can be observed, which was already described for FCS-generated moDCs earlier (34, 35). As we previously observed for DCs derived from the monocytic cell line, THP-1 (36), FCS-generated DCs possess high productive infection. FCS pre-coated wells resulted in moDCs with elevated levels of virus particle generation in comparison to uncoated hPL-treated moDCs, suggesting a beneficial effect of FCS components for progressive infection.

Confocal microscopic analysis presents a different pattern of CD11c and HLA-DR expression with a higher presence of microtubule connections between cells for virus transmission in FCS-moDCs. mCherry HIV signal can be observed at higher density cell clusters specifically, supporting the sentinel role of DCs (37). The amounts of cells detected via the automated quantification tool in the harmony software shows an interesting decline of DCs when comparing the uncoated to the FCS pre-coated hPL-generated cells with solely FCS-based cells showing superior cell numbers. The resulting quantities of virus particles normalized to the cell numbers present similar relations between HIV-1 and HIV-C among the conditions with FCS application being more beneficial for virus generation. However, it cannot be excluded that the density of DCs enhanced cell-to-cell transmission potential via numerous microtubules (38). In conclusion our data reveal a thorough characterization of DCs cultured in various animal-derived and -free sera in their differentiation, maturation and infection potential. We here describe an animal component-free *in vitro* model to generate primary CD11c^+^CD209^+^-moDCs by use of hPL, allowing to successfully study virus-DC transmission and interactions at mucosal surfaces. The use of an hPL-based, xeno-free model enhances the translational relevance of research on dendritic cell (DC)-mediated HIV interactions by providing a human-derived, serum-free environment that more closely resembles physiological conditions than conventional FCS-containing systems. This model is particularly useful in preclinical settings, where reducing xenogeneic influences is essential for studying immune cell differentiation, function, and HIV susceptibility under human-relevant conditions. While hPL is not autologous and therefore not applicable for generating clinical-grade, patient-specific immunotherapies, it offers a scalable and ethically favorable alternative for the development and optimization of HIV vaccine candidates and *in vitro* immunological assays. Potential clinical applications include improved testing of DC-targeted vaccine strategies or evaluation of innate immune responses in HIV-infected individuals. Nevertheless, several limitations of the hPL model must be acknowledged-broader adoption of this model may face challenges, including inter-batch variability of hPL, regulatory hurdles for standardization, and the need for harmonized protocols across laboratories. Moreover, as with FCS-DCs there might be differences to the *in vivo* interactions due to not fully replicating the complex cellular and tissue microenvironments of human mucosa or lymphoid organs. Finally, the use of expired platelet concentrates, while ethically advantageous, may not fully reflect the functional composition of platelets in circulation, potentially influencing the cytokine and growth factor profile of the lysate. These factors should be considered when extrapolating our findings to *in vivo* or clinical scenarios. Nevertheless, the use of hPL in our model system may also reflect certain aspects of the *in vivo* environment relevant to HIV and HIV-associated comorbidities. Platelet activation and lysis are commonly observed in people living with HIV, even under suppressive antiretroviral therapy (ART), and are further exacerbated by co-infections such as HCV or CMV (39). HIV itself has been shown to induce platelet activation and promote interactions between platelets and immune cells, contributing to systemic inflammation, endothelial dysfunction, and immune dysregulation (40, 41). In this context, lysed platelets can release a range of pro-inflammatory mediators, damage-associated molecular patterns (DAMPs), and microparticles that may act as endogenous danger signals, influencing dendritic cell maturation and function (42, 43). Therefore, while hPL is not a direct surrogate for autologous plasma or tissue environments, its use *in vitro* may approximate conditions of heightened platelet turnover, immune activation, and cytokine release seen *in vivo* during chronic HIV infection. Compared to other xeno-free supplements, hPL may thus provide a more immunologically relevant stimulus, capturing both supportive and inflammatory cues that influence dendritic cell–virus interactions.

## Materials and methods

### Human platelet lysate

Human platelet lysate (hPL) used in this study was obtained from the Blood Bank of the University Hospital Innsbruck. The hPL was produced under standardized and GMP-compliant conditions as part of a long-standing collaborative effort involving the Innsbruck blood bank and the Gstraunthaler group. The production protocol ensures batch-to-batch consistency and includes stringent quality control procedures. Detailed compositional analyses of this hPL product—including levels of platelet-derived cytokines, coagulation factors, and growth factors such as TGF-β—have been previously published (44). In particular, key bioactive components were quantified and shown to support robust cell growth in serum-free culture systems (45). The same hPL formulation has also been referenced in international consensus efforts for GMP-grade hPL production and application in cell therapy and *in vitro* research (46). Based on this extensive prior characterization, no additional quantification of individual hPL components was performed within the present study.

### Platelet collection and hPL preparation

Platelet concentrates (PLTs) used for hPL production were obtained either from pooled buffy coats or via apheresis from single donors. All procedures were carried out at the Central Institute for Blood Transfusion and Immunology of the Tirol Kliniken in Innsbruck under certified standard operating procedures and in accordance with Austrian national regulations. Pooled PLTs were prepared from six buffy coats using the CompoStop Flex Triple, T&B Set (Fresenius Kabi, REF: FT52600), centrifuged at 560 × g for 9 minutes and 10 seconds at 20°C, and leukocyte-depleted using the Macopress Smarter system (Fresenius Kabi). Apheresis PLTs were collected using either the Trima Accel (Terumo BCT, Zaventem, Belgium) or Amicus (Fresenius Kabi) cell separation platforms. All PLTs were suspended in 35% plasma and 65% platelet additive solution—either InterSol (PAS-C, REF: RGR8109B, Fresenius Kabi) or SSP+ (PAS-E, REF: SSP2130U, Macopharma, France). Pathogen inactivation was performed using the INTERCEPT blood system (Cerus Europe, Amersfoort, Netherlands) following the manufacturer’s protocol, as described previously (47). All products were tested for HIV, HCV, HBV, and a subset for bacterial contamination using the BACT/ALERT 3D system (bioMérieux, France). Only expired PLTs (stored for 7 days at 22°C) were used for the production of human platelet lysate (hPL). Platelets were lysed by three freeze (–80°C) and thaw (37°C) cycles. To minimize donor-to-donor variability, five platelet concentrates were pooled for each hPL batch. As each concentrate was derived from six buffy coats, this corresponds to a total donor pool of 30 individuals per batch. This approach follows published recommendations suggesting a minimum of four PLT units per batch to ensure reproducibility and reduce inter-individual variation (48). The pooled lysate was centrifuged at 5,500 × g for 15 minutes at 22°C, and the supernatant was sterile filtered using 0.2 µm filters (Nalgene Rapid-Flow, Cat. No. 565-0020, Thermo Fisher Scientific) under aseptic conditions in a class A laminar flow hood. Final hPL products were stored at –20°C until use. All PLT products were originally manufactured for clinical use under a license from the Austrian Agency for Health and Food Safety (AGES) and in compliance with ISO 9001:2015 quality standards.

### Virus

The viruses used for the experiments in this project are produced in house in batches and listed in **Supplementary Table 1**. Supernatants resulting from the transfection of HEK293 cells with plasmids, provided by Prof. Thomas J Hope from the Northwestern University in Chicago, are concentrated via ultracentrifugation at 20,000 rpm for 90 min at 4°C. An aliquot of each virus produced is inoculated with normal human serum that was stored at -80°C at maximum for 6 months to retain complement activity to generate complement-opsonized virus stocks. The concentrations of the viruses are analyzed via p24 ELISA. Their opsonization pattern is checked via virus capture assay and the infectivity (TCID50) is deduced by application of infection assays. The m-cherry tagged HIV-1/HIV-C are produced via calciumphosphate-mediated transfection of HEK293T/17 cells with R9BaL and mCherry proviral plasmids.

### Opsonization of HIV-1

Ultracentrifuged, concentrated virus was opsonized in 200µl with medium alone (HIV), or normal human serum (NHS) as active complement source (HIV-C). Subsequent to opsonization, the different preparations were washed using 1 ml RPMI1640 without supplements (RPMI) and the virus was pelleted by ultracentrifugation. The virus was re-suspended in 200µl RPMI, aliquoted and the presence of C3 fragments or IgGs on the viral surface was confirmed by a virus capture assay (VCA) as described (34, 35).

### Generation of human monocyte-derived DCs

Peripheral blood of healthy donors was obtained by the Central Institute for Blood Transfusion and Immunological Department, Innsbruck, Austria (EC1166/2018). Monocyte isolation was performed as previously described (19, 34). Briefly, PBMCs were isolated by use of a density gradient centrifugation using a Ficoll Paque Premium (GE Healthcare) gradient. Classical CD14+ monocytes were isolated using anti-human CD14 Magnetic Beads (BD). Only cells >98% purity were considered for further analysis. For efficient generation of immature DCs (iDCs), 1x10^6^ monocytes/mL were seeded and stimulated with IL-4 (200 U/ml) and GM-CSF (250 U/ml) for 5 days. Cells were either cultured in RPMI media containing 10% FCS or 5% hPL. For FCS-pre-coated human platelet lysate (chPL) DCs, wells were pre-incubated with 1% FCS (1h, 37°C) before cultivation in 5%hPL. L-glutamine was added in all culture conditions.

### Stimulation and infection of DCs

Dendritic cells were stimulated in U-bottom 96-well plates. The assay was performed in triplicates. Cells were either treated with 100 ng/mL LPS or 50 ng/100 µL HIV. RPMI media was used as a blank control. The cells were incubated for 24h at 37°C, 5% CO2. The supernatants were harvested for p24 ELISA analysis.

### p24 ELISA

p24 ELISA was performed as described previously (49). All antibodies were kindly provided by Polymun Scientific, Vienna, Austria.

### Confocal microscopy

Cells are prepared as described above. The assay was performed on 96-well Perkin Elmer LLC CellCarrier-96 Ultra Microplates. To analyze infection, mCherry-tagged non- and complement-opsonized HIV-1 preparations were used (HIV, HIV-C). Prior to fixation, the cell surface was in additionally stained using CD11c Alexa 647 and HLA-DR Alexa 488. The next day, an intracellular staining was performed using an antibody mix of CD11c Alexa 647, HLA-DR Alexa 488 and Hoechst 33342. Plates were imaged on the Operetta^®^ CLS™ high content screening system (Perkin Elmer) and spots, XYZ and 3D analyses were calculated using the Harmony™ software.

### Flow cytometry

For flow cytometry analysis, cells were stained and analyzed as previously described and are routinely tested in the laboratory using a viability dye, e.g. Ghost Dye Violet 540 Fixable Viability Dye (Cell Signaling Technology) or fixable Viability Dye (Thermo Fisher Scientific) (19, 50). In brief, cells were harvested and washed with FACS wash (0.5% BSA, 0.1% sodium azide in PBS). Prior to antibody or live/dead staining, Fc receptors were blocked, if indirect staining was performed. All samples were fixed in 4% FACS-fixation buffer, stained according to standard operating procedures and analyzed using BD FACS VERSE™ flow cytometer and the Flowlogic™ software. All antibodies used are listed in **Supplementary Table 2**.

### Statistical analysis

Differences between 2 groups were analyzed using GraphPad Prism software (Version 8.0.1) (GraphPad Software Inc.) with the unpaired Student t test (2-tailed). For multiparameter comparisons, values from the experiments were analyzed by use of One-way ANOVA, corrected with Tukey multiple comparison testing. Tests used are indicated in the Figure legends.

## Data Availability

The original contributions presented in the study are included in the article/**Supplementary Material**. Further inquiries can be directed to the corresponding author.
