# Supplementary material for: Serum matters: human platelet lysate enables physiological modeling of HIV-1 infection in dendritic cells
Source: Front Immunol. 2025 Sep 17;16:1661425. doi: 10.3389/fimmu.2025.1661425 (PMC12484019; doi:10.3389/fimmu.2025.1661425)
Supplement: Supplementary file 1 [file Table1.docx]

**Supplementary tables**

**Supplementary Table 1**

| **strain** | **specification** | **tropism** |
| --- | --- | --- |
| YU-2(B) | complement-opsonized HIV-1 (HIV-C) | CCR5 |
| YU-2(B) | non-opsonized HIV-1 (medium control) | CCR5 |
| R9BaL | complement- opsonized HIV-1 (HIV-C) | CCR5 |
| R9BaL | non-opsonized HIV-1 (medium control) | CCR5 |
| R9BaL | mCherry tagged HIV-1 | CCR5 |
| R9BaL | mCherry tagged complement- opsonized HIV-1 (HIV-C) | CCR5 |

**Supplementary Table 2**

| **Antibody / Reagent** | **manufacturer** | **catalogue number** |
| --- | --- | --- |
| CD11c FITC | BioLegend | 301604 |
| CD11c Alexa 647 | BioLegend | 301620 |
| CD11c BV421 | BD Bioscience | 562561 |
| CD14 PE | BioLegend | 301806 |
| CD83 PE | BioLegend | 305308 |
| CD86 BrilliantViolet510 | BioLegend | 305432 |
| DC-SIGN APC | BioLegend | 330108 |
| DC-SIGN PerCP-Cy5.5 | BioLegend | 330110 |
| HLA-DR Alexa 488 | BioLegend | 307620 |
| HLA-DR BV421 | BioLegend | 307636 |
| Live/Dead Fixable Far Red | Thermo Fisher Scientific | L34973 |
| Ghost Dye UV 450 fixable | Cell Signaling Technologies | 80862 |
| M01 antibody | Polymun Scientific GmbH | AB005 |
| 37G12 monoclonal antibody | Polymun Scientific GmbH | AB006 |
